# Manipulating Bimetallic Nanostructures With Tunable Localized Surface Plasmon Resonance and Their Applications for Sensing

**DOI:** 10.3389/fchem.2020.00411

**Published:** 2020-05-19

**Authors:** Yuanhong Min, Yi Wang

**Affiliations:** Chongqing Key Laboratory of Green Synthesis and Applications, College of Chemistry, Chongqing Normal University, Chongqing, China

**Keywords:** metal nanoparticles, shape control, galvanic replacement, seed-mediated growth, oxidative etching, colorimetric detection

## Abstract

Metal nanocrystals with well-controlled shape and unique localized surface plasmon resonance (LSPR) properties have attracted tremendous attention in both fundamental studies and applications. Compared with monometallic counterparts, bimetallic nanocrystals endow scientists with more opportunities to precisely tailor their LSPR and thus achieve excellent performances for various purposes. The aim of this mini review is to present the recent process in manipulating bimetallic nanostructures with tunable LSPR and their applications for sensing. We first highlight several significant strategies in controlling the elemental ratio and spatial arrangement of bimetallic nanocrystals, followed by discussing on the relationship between their composition/morphology and LSPR properties. We then focus on the plasmonic sensors based on the LSPR peak shift, which can be well-controlled by seed-mediated growth and selective etching. This review provides insights of understanding the “rules” involving in the formation of bimetallic nanocrystals with different structures and desired LSPR properties, and also forecasts the development directions of plasmonic sensors in the future.

## Introduction

Metal nanocrystals with fascinating localized surface plasmon resonance (LSPR) properties have received increasing attention over the past several decades. They have played important roles in a variety of different areas such as sensing, imaging, photocatalysis, photovoltaic devices, and photothermal therapy (Anker et al., 2008; Jain et al., 2008; Rycenga et al., 2011; Wang and Asrtuc, 2014; Li X. et al., 2016). The LSPR of a nanosized metal particle arises from the collective oscillation of conduction electrons in the particle which is excited by electromagnetic radiation. When the incident light matches the resonance wavelength of the metal nanoparticles which are much smaller than the incident wavelength, the photons are able to be absorbed and a localized surface plasmon will be stimulated (Haes and Van Duyne, 2004; Willets and Van Duyne, 2007). Among the numerous metallic elements, only Au and Ag nanoparticles are widely used as plasmonic materials since their LSPR absorption and scattering are located in the visible light region, as well as their stability and easy preparation. Many other metals such as Pd, Hg, Pb, Bi, In, and Sn can also generate LSPR, however, the study of these nanoparticles is hampered by their relatively weak LSPR in ultraviolet region, high susceptibility to oxidation, or the difficulty in shape-controlled synthesis (Xia et al., 2009; Li et al., 2018, 2019b,c; Chen et al., 2019). Except for composition, the LSPR properties of a metal nanostructure are also critically dependent on its physical parameters including size, shape, and internal structure (Murphy et al., 2005; Jain et al., 2007; Tao et al., 2008). For example, the LSPR peak of Au nanoparticles split into two (transverse and longitudinal LSPR) when they transform from zero-dimensional nanospheres into one-dimensional nanorods (Chen et al., 2013). Pd nanoparticles exhibit very weak and broad LSPR in ultraviolet and visible regions (Xiong and Xia, 2007), however, ultrathin Pd nanosheets display well-defined LSPR peaks in the near-infrared region (Huang et al., 2011). Thus, the number of resonant modes or LSPR peak position can be tailored by manipulating any one of these parameters or a combination of them.

Compared to the nanocrystals composed of only one metal, bimetallic nanostructures are more powerful in regulating the LSPR properties (Cortie and McDonagh, 2011; Mayer and Hafner, 2011; DeSantis et al., 2013). On the one hand, the composition of bimetallic nanocrystals (i.e., the ratio of two different metals) can be controlled to achieve the LSPR regulation since the LSPR of different metals locates in different wavelength regions. For example, the LSPR peak of 20-nm Au nanoparticles usually appears around 520 nm and the solution displays a wine red color. However, Ag nanoparticles in the same size usually show the LSPR peak at ~400 nm and a bright yellow solution (Haes and Van Duyne, 2004). Even so, it is inadequate to just use “composition” to describe bimetallic nanocrystals. On the other hand, the LSPR of bimetallic nanostructures is also dependent on their morphology. Namely, the number, position and profile of the LSPR peak are highly sensitive to the spatial arrangement and atomic ordering of the two different types of metal atoms. For instance, although the molar ratio of Ag/Au kept the same, fully alloyed Ag-Au nanoparticles exhibited different LSPR peak position from the Au@Ag core-shell nanoparticles, together with much narrower bandwidth (Gao C. et al., 2014). Single-, double-, and triple-walled nanotubes made of Au-Ag alloy also displayed quite different LSPR spectra involving both peak position and profile (Sun and Xia, 2004a).

Thanks to a great deal of effort from many research groups, a large number of bimetallic nanocrystals with well-defined structures and tunable LSPR have been achieved (Hou et al., 2013; Gilroy et al., 2016). The most commonly approaches to the synthesis of bimetallic nanostructures in solution phase are introduced in this review, including co-reduction, seed-mediated growth, and galvanic replacement reaction. Owing to the extensive studies of Au-Ag nanocrystals referring to their synthetic strategies and LSPR-based applications, we will focus on the review of Au-Ag bimetallic nanocrystals with well-defined structures and tunable LSPR. Besides, combined with another plasmonic metal of Cu, Au-Cu, and Ag-Cu nanocrystals can also give rise to strong LSPR. Other metallic elements (e.g., Pd or Pt) are only mentioned in somewhere because they usually lead to drastic dampening or quenching the LSPR of another plasmonic metal. Benefiting from the sensitivity of plasmon resonance toward changes in the local dielectric environment, the peak position and profile of LSPR will be drastically altered when any object gets close to the nanoparticles. Furthermore, the LSPR peak shift will be more sensitive once the object can directly transform the composition or structure of the nanoparticles themselves. Based on these findings, a large number of sensors have been developed by controlled management of the LSPR absorption or scattering of the metal nanocrystals. In this review, we will focus on the plasmonic sensors based on the LSPR peak shift, which can be well-controlled by manipulating the composition and morphology of bimetallic nanostructures. Aggregation-induced LSPR alteration and its related analytical methods will not be concerned herein since bimetallic nanoparticles do not take obvious advantages over single-metallic nanoparticles in this aspect. In addition, the aggregation degree of nanoparticles can hardly be precisely controlled because a number of parameters are able to affect this process.

## Strategies to Manipulation of Bimetallic Nanostructures

**Figure 1:**
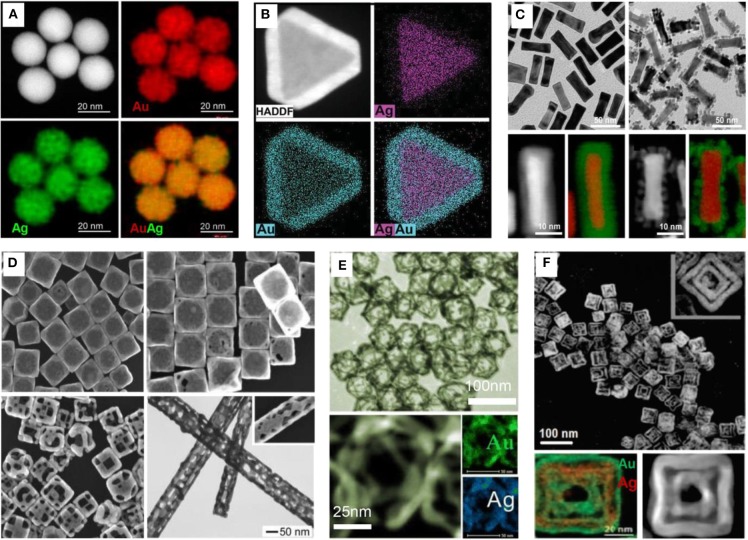
Bimetallic nanocrystals with various shapes and structures. **(A)** Alloyed Au-Ag nanospheres. Reproduced from Gao C. et al. (2014). with permission from American Chemical Society. **(B)** Core-shell Ag@Au nanoplates. Reproduced from Liu H. et al. (2015); Liu W. et al. (2015) with permission from Wiley-VCH Verlag GmbH & Co. KGaA, Weinheim. **(C)** Core-shell Au@Pd nanorods. Reproduced from Chen et al. (2012) with permission from American Chemical Society. **(D)** Hollow Au-Ag nanoboxes, nanocages, nanoframes, and nanotubes. Reproduced from Sun and Xia (2004b) with permission from American Chemical Society. **(E)** Octahedral Au-Ag nanoframes. Reproduced from Hong et al. (2012) with permission from American Chemical Society. **(F)** Double-walled Au-Ag nanoboxes. Reproduced from González et al. (2011) with permission from American Association for the Advancement of Science.

LaMer model is widely used to describe the nucleation and growth mechanism of metal nanocrystals in solution phase (Xia et al., 2009). Metal precursors are firstly transformed into metal atoms via reduction or decomposition, which start to aggregate into small clusters (i.e., nuclei) once their concentration has reached the point of supersaturation. Then, the nuclei grow up to seeds and thereafter formation of shape-controlled nanocrystals under the control of thermodynamics and kinetics. In this section, considering the repeatability and stability of the bimetallic nanocrystals in applications, we focus on the controllable and universal syntheses including co-reduction, seed-mediated growth, and galvanic replacement. Thanks to a great effort of many research groups, a large number of bimetallic nanocrystals with different architectures are available (Sun and Xia, 2004b; González et al., 2011; Chen et al., 2012; Hong et al., 2012; Gao C. et al., 2014; Liu H. et al., 2015; Liu W. et al., 2015) (Figure 1).

Co-reduction is the most straightforward way to synthesize bimetallic nanocrystals, where two metal precursors are simultaneously reduced into metal atoms and then nucleation and growth together. Different bimetallic nanostructures can be generated by varying experimental parameters such as the reduction potentials of metal precursors, strength of reducing agent, coordination ligands, reaction temperature, and capping agent. It should be pointed out that the reduction potential of metal ions involved plays a pivotal role in their formation. Generally, metal ions with similar reduction potential should be selected to get alloyed structures by co-reduction. For example, Pd^2+^ and Pt^2+^ can be readily coreduced to form Pd–Pt alloyed nanocrystals since they have adjacent reduction potentials (Pd^2+^/Pd, +0.9 V; Pt^2+^/Pt, +1.18 V) (Liu Y. et al., 2011a,b; Liu N. et al., 2011c). If there is a large gap of the reduction potentials between two metal ions (e.g., Au^3+^/Au, +1.5 V; Ag^+^/Ag, +0.8 V), the more noble metal will be reduced first followed by the other. This problem might be overcome by varying the molar ratio of the two metal precursors, as well as using a very strong reductant and introducing appropriate coordination ligands to achieve the synchronized reduction of metal ions (Wang et al., 2009, 2015a; Kim et al., 2014; Lohse et al., 2014).

Seed-mediated growth is a powerful and versatile means to synthesize bimetallic nanocrystals with core-shell structures. In this process, preformed metal seeds with well-defined characteristics serve as primary sites for the deposition of newly formed atoms that generated from reduction or decomposition of another metal precursor (Xia et al., 2016). The first example of seed-mediated growth for bimetallic nanostructure was reported at 2002, where Ag nanowires were prepared from the Pt seeds (Sun et al., 2002b). Thereafter, this approach was further expanded to other bimetallic systems such as Pt-Pd, Au-Ag, Au-Pd, Pd-Cu, Pd-Ag, Pd-Rh, Pd-Ir, and so on (Habas et al., 2007; Ma et al., 2010; Jin et al., 2012; Li et al., 2012; Zeng et al., 2012; Xie et al., 2013; Xia et al., 2014). The morphologies of the bimetallic nanocrystals obtained by this method were also expanded from core-shell structure to alloyed, core-frame, branched, and heterostructured ones (DeSantis and Skrabalak, 2012, 2013; Xie et al., 2012b; DeSantis et al., 2013; Gao C. et al., 2014; Bai et al., 2017). Compared with co-reduction route, seed-mediated growth is readily to elaborately engineer the morphology and thus properties of the bimetallic nanocrystals. Moreover, it can also serve as a model system to elucidate the mechanisms involved in nanocrystal synthesis since the nucleation and growth processes are divided in this approach.

Galvanic replacement is an electrochemical process that can fabricate complex hollow bimetallic nanostructures with well-controlled properties. It usually occurs in the system involving the oxidation of one metal (acts as a sacrificial template) by the ions of another metal having a higher reduction potential (Xia et al., 2013). As a result, the template will be oxidized and gradually dissolved into the solution while the second metal ions will be reduced into metal atoms and plated onto the outer surface of the template. Xia et al. conducted the galvanic replacement reaction on nanocrystals for the first time in 2002, where Ag-Au hollow nanostructures were achieved using Ag nanoparticles as sacrificial templates (Sun and Xia, 2002; Sun et al., 2002a). Thereafter, a lot of research groups extended the galvanic replacement to many other bimetallic or trimetallic nanocrystals (e.g., Ag-Pd, Ag-Pt, Pd-Pt, and Pd-Au-Cu) as well as more complex structures (e.g., nanoframe, yolk–shell nanocage, nanorattle, and multiple-walled nanoshells/nanotubes) (Métraux et al., 2003; Sun and Xia, 2004a; Chen et al., 2005; González et al., 2011; Zhang et al., 2011; Xie et al., 2012a), and also systematically studied on the mechanism in this process (Sun and Xia, 2004b; Sun et al., 2004; Chen et al., 2006; Skrabalak et al., 2007b). Galvanic replacement provides a simple and maneuverable approach for precisely tuning the LSPR peaks of plasmonic metal nanostructures by adjusting the amount of another metal ion relative to the template (Skrabalak et al., 2007a, 2008; Wang et al., 2013a,b; Xia et al., 2020). The limitation of this approach is no more than the requirement of a favorable difference in the reduction potentials of the two metals.

## Effect of Composition and Morphology on LSPR of Bimetallic Nanostructures

The LSPR peak position and profile of metal nanocrystals is determined by a number of parameters including composition, size, shape, internal structure, and the dielectric constant of the surrounding environment. For bimetallic nanostructures, one of the most important factors that can profoundly impact on the LSPR properties is their composition, which involves both the elemental ratio and atomic spatial distribution of two different metals. El-Sayed et al. synthesized Au–Ag alloy nanoparticles with varying Au/Ag mole fractions by coreduction of HAuCl_4_ and AgNO_3_ with sodium citrate. They observed a linear blue shift of the LSPR peak with increasing Ag content while an exponential decrease of the extinction coefficient with increasing Au content (Link et al., 1999). Other groups further confirmed this finding (Mallin and Murphy, 2002; Moskovits et al., 2002; Kim et al., 2003; Shore et al., 2010). Similarly, the Au–Cu and Ag–Cu alloy nanocrystals also displayed varying LSPR according to the stoichiometry of the different two elements in a particle (Smetana et al., 2006; Cattaruzza et al., 2007; Motl et al., 2010; Kim et al., 2014; Hajfathalian et al., 2015). As compared to Au–Ag nanoparticles, the syntheses and applications of Cu-involved bimetallic nanocrystals were limited due to the chemical instability of Cu. On the other side, when a plasmonic metal combined with Pd or Pt to generate an alloyed nanocrystal (e.g., Ag–Pd, Ag–Pt, and Au–Pt), the initial LSPR could be inhibited or completely quenched (Zhang S. et al., 2013; Cargnello et al., 2015). The quench of LSPR might be attributed to a different electronic structure of the two metals and/or to the presence of inhomogeneous doping/alloying.

The spatial distribution of different types of atoms in a bimetallic nanocrystal also significantly affects the LSPR. For instance, a number of studies have found that bimetallic heterostructures exhibit more complex LSPR behavior compared to the alloys containing the same metals. Taking core-shell nanostructures as an example, their LSPR is mainly determined by both the element and thickness of the shell, while the contribution of the core to LSPR is relatively limited. For example, Xia et al. synthesized Au@Ag core–shell nanocubes and found that the LSPR rapidly blue-shifted as the thickness of Ag shell increased. The LSPR signal of Au core was completely masked by that of Ag once the Ag shell exceeded 3 nm (Ma et al., 2010). Consistent conclusions were also obtained by other groups, including the case that switching Ag as the core and Au as the shell (Zhang et al., 2008; Kahraman et al., 2009; Zhang W. et al., 2013; Tsao et al., 2014; Liu H. et al., 2015; Liu W. et al., 2015). When other metals with no LSPR in the visible region (e.g., Pd or Pt) served as the core and plasmonic metals (e.g., Au, Ag, or Cu) served as the shell, the LSPR properties of the core-shell nanoparticle were also dominated by its shell. The studies on many cases of Pd–Ag, Pd–Ag, Pt–Au, and Pd–Cu bimetallic nanocrystals provided direct experimental evidences (Henglein, 2000; Lahiri et al., 2005; Jin et al., 2012). In contrast, coating a plasmonic nanocrystal (e.g., Au, Ag, or Cu) with Pd or Pt resulted in the attenuation even quenching of the initial LSPR signal (Mandal et al., 2004; Khanal and Zubarev, 2009; Chen et al., 2012; Chiu et al., 2014).

Similar to monometallic nanocrystals, the morphology (e.g., geometrical shape, internal structure, and surface state) of bimetallic nanostructures is also crucial to their LSPR properties. Compared to spherical-like nanoparticles, the anisotropic nanostructures can exhibit more than one LSPR peak as the number of peaks directly corresponds to the number of polarized ways (Gilroy et al., 2016). Taking nanorod as a typical example, it shows transverse and longitudinal resonant modes at shorter and longer wavelengths, respectively, corresponding to the polarization of free electrons along the short and long axis of the nanorod. Each of the two modes and thus the wavelength of LSPR peaks can also be readily tuned through adjusting the aspect ratio of the rods (i.e., increase their thickness or length). Yin et al. synthesized fully alloyed Ag-Ag nanospheres and nanorods by controlled high-temperature annealing in confined spaces. The nanospheres exhibit only one LSPR peak, while the nanorods have both transverse and longitudinal LSPR peaks (Gao C. et al., 2014; Bai et al., 2017). Wang et al. prepared Au@Ag core–shell nanorods by seeded growth of Ag mainly on the sides of Au nanorods. Along with increasing the amount of Ag precursor, Au@Ag nanorods with decreased aspect ratio were obtained. They found that the two LSPR bands of the seeds (Au nanorods) transformed into four bands when the Au@Ag core–shell nanorods were formed, where the lowest- and second-lowest-energy peaks belong to the longitudinal and transverse dipolar plasmon modes, respectively, and the two highest-energy peaks are ascribed to octupolar plasmon modes (Jiang et al., 2012). Subsequently, Liz-Marzán and co-workers achieved the synthesis of Ag–Au–Ag nanorods by depositing Ag at both ends of each Au nanorod. They could precisely control over the length of the bimetallic nanorods from ~210 nm to several micrometers and meanwhile kept their thickness almost unchanged. As a result, the longitudinal LSPR peak could be tuned in the range of 1,100–2,250 nm while the transverse peak stayed relatively constant (Mayer et al., 2015). The case of anisotropic Ag–Au bimetallic nanoplates also demonstrated the correlation of morphology and LSPR, where the shift direction of LSPR band was dependent on the deposition of Au on the {111} or {100} facets (Liu H. et al., 2015; Liu W. et al., 2015).

## Plasmonic Sensors Based on LSPR Peak Shift of Bimetallic Nanostructures

The LSPR of metal nanoparticles are sensitive to their local dielectric environment. When other substances are brought into the proximity of metal nanoparticles to alter their surrounding environment, their LSPR properties will change. McFarland and Van Duyne found that the LSPR scattering peak of individual Ag nanoparticles greatly shifted when they were in solvents with different refractive indexes. Based on this concept, an optical sensor for 1-hexadecanethiol detection was developed with zeptomole sensitivity (McFarland and Van Duyne, 2003). Huang et al. also proved this conclusion in Au nanoparticles (Liu Y. et al., 2011a,b; Liu N. et al., 2011c; Liu and Huang, 2013a). Another typical example of this concept is the LSPR hydrogen sensor. Owing to the hydrogen atoms can be reversibly absorbed into the lattice of Pd nanocrystals (Baldi et al., 2014), Alivisatos et al. designed a model system consist of a triangular Au nanoplate in the proximity of Pd nanoparticles to sensing H_2_. The sensing was achieved based on the hydrogen-induced change in dielectric function of the Pd nanoparticles, which further decreased the near-field enhancement of proximal Au nanoplates (Liu Y. et al., 2011a,b; Liu N. et al., 2011c). Later studies on the adsorption/desorption behavior of H_2_ on bimetallic single Au@Pd core–shell naoparticles further demonstrated that the LSPR of the Au core was sensitive to the adsorption and desorption of H_2_ on the surface of the Pd shell, which enable fast sensing of H_2_ at low concentrations (Tang et al., 2011). Recently, Han et al. found that colloidal clusters of Au@Pd core–shell nanoparticles exhibited remarkably enhanced sensitivity for H_2_ detection compared to their Au@Pd nanoparticle counterparts. Simulations verified that LSPR-induced intense near fields are localized around their interparticle gaps (Wy et al., 2018). Although great achievements had been made, almost of these studies could only record the variation of LSPR signal on single-particle level. In other words, the changes in the collective LSPR properties of nanoparticles are hard to detect by this way. Moreover, extension of this strategy for sensing applications is also limited due to the specificity between Pd nanocrystals and H_2_.

**Figure 2:**
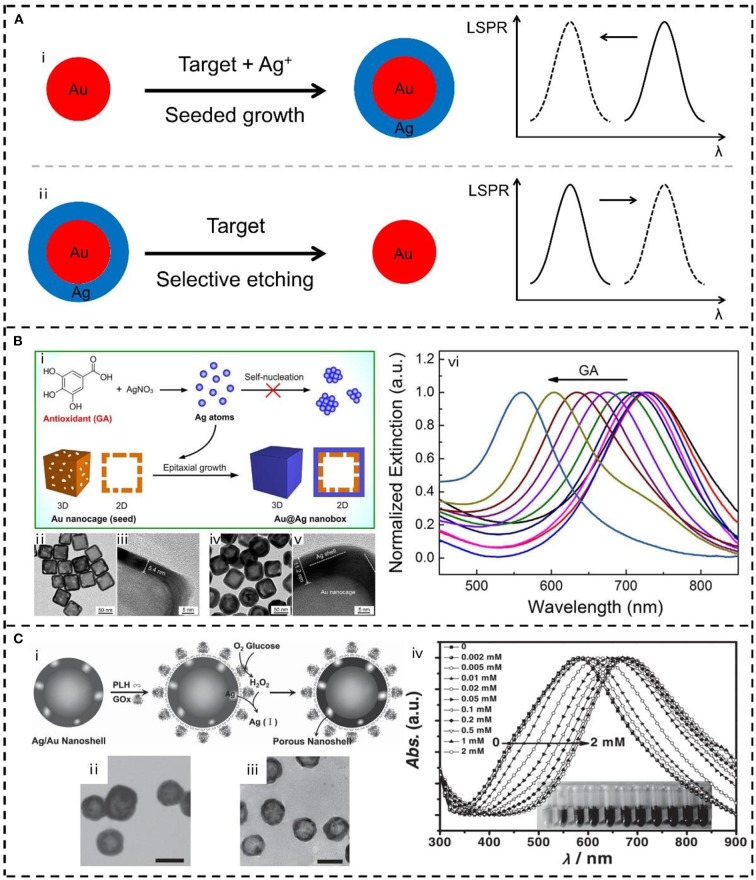
**(A)** Schematic representation of the plasmonic sensing strategies based on LSPR peak shift of bimetallic nanostructures: (i) seeded growth, and (ii) selective etching. **(B)** Plasmonic sensor based on the seed-mediated growth of Au@Ag nanobox: (i) Schematic illustration, (ii–v) transmission electron microscope (TEM) images and (vi) spectral evolutions of the LSPR sensor for antioxidants detection. Reproduced from Wang et al. (2018a) with permission from Elsevier. **(C)** Plasmonic sensor based on the selective etching of Ag in Au-Ag nanoshells: (i) Schematic illustration, (ii, iii) TEM images and (iv) spectral evolutions of the LSPR sensor for glucose detection. Reproduced from He et al. (2012) with permission from Wiley-VCH Verlag GmbH & Co. KGaA, Weinheim.

Alternatively, record of collective LSPR signal of bimetallic nanoparticles for colorimetric detection is more convenient, practical, and cost-effective than that of monitoring LSPR on single particles. Figure 2A represents two typical plasmonic sensing strategies based on LSPR peak shift of bimetallic nanostructures, namely, seed-mediated growth and selective etching. Seed-mediated growth can generate bimetallic nanocrystals with controlled composition and structure, where the LSPR is facile to be manipulated for plasmonic sensing. For example, Stevens et al. employed an enzyme to control the nucleation rate of Ag on plasmonic Au nanostars and thus developed an LSPR sensor for the detection of cancer biomarker (Rodríguez-Lorenzo et al., 2012). Specifically, Ag ions were able to be reduced to Ag atoms by H_2_O_2_ which generated from the enzymatic reaction of GOx. When the concentration of GOx was low, epitaxial growth of Ag on Au nanostars occurred due to the relatively low reduction kinetics, leading to an obvious blue-shift of the LSPR of the nanostars. When the concentration of GOx was high, self-nucleation of Ag in solution was instead of the epitaxial growth on Au nanostars, which displayed a small LSPR shift. It should be highlighted that ultrasensitive detection was achieved (down to 10^−18^ g mL^−1^) since the LSPR shift was oppositely proportional to the concentration of analyte. Recently, our group also developed a series of sensors for the detection of antioxidants in food and cosmetics based on the concept of seed-mediated growth. Employing either hollow Au nanocages or solid Au nanorods as the seeds to grow Ag shell, we observed continuous blue shift of their LSPR as increasing the concentration of antioxidants (Figure 2B) (Wang J. et al., 2016; Wang Y. et al., 2016; Li L. et al., 2018; Wang et al., 2018a,b). It means that one can precisely tailor the LSPR of Au@Ag core-shell nanostructures for sensing through the controlled seeded growth, in which the concentration of Ag precursor and the reduction kinetics are both important. Similar approaches were also reported by several other groups, which had been achieved in the detection of perishable products (Zhang C. et al., 2013), phosphatase activity (Gao Z. et al., 2014), and influenza virus (Xu et al., 2017). Except for the studies on the collective LSPR by spectroscopy, individual bimetallic plasmonic nanoparticles were also monitored by dark-field microscopy for real-time sensing. For example, *in situ* formation of alloyed bimetallic nanoparticles (e.g., Ag-Hg and Au-Hg) was successfully monitored in real-time by dark-field scattering microscopy (Liu and Huang, 2013b; Wang J. et al., 2016; Wang Y. et al., 2016). Generation of Au@Ag “nanosnowman” by heterogeneous nucleation was recorded on single-particle level for ultra-sensitive microRNA detection (Zhao et al., 2019). Also, the dark-field scattering microscopy and spectroscopy of single bimetallic plasmonic nanoparticles had been extended to various targets detection (Mashtalir et al., 2013; Hao et al., 2014; Lei et al., 2015).

In the opposite direction of seed-mediated growth, selective etching of the metallic nanocrystals is also an effective approach to LSPR adjustment (Wang et al., 2015b; Zhang et al., 2020). A metallic component from the preformed bimetallic nanocrystals is especially suited to fabricate plasmonic sensors benefiting from the difference between two metals. Along with the target-induced etching proceeds, element ratio and morphology (e.g., formation of hollow or porous structures) of the bimetallic nanocrystals both changes, where tunable LSPR can be managed as sensing signal. Jin et al. constructed GOx enzyme-immobilized Ag-Au bimetallic hollow nanoshells for glucose detection through this idea (He et al., 2013; Chen et al., 2015). As shown in Figure 2C, enzymatic oxidation of glucose in air produced H_2_O_2_, which could selectively dissolve Ag from the preformed Ag/Au bimetallic nanoshells. In that case, porous nanoshells with decreased Ag/Au ratio were obtained, resulting in the red shift of their LSPR peak. This kind of bimetallic nanoshells was also applied to *in vitro* visual discriminate and photothermal killing of cancer cells (Jin, 2014; Chen et al., 2015). Xia et al. fulfilled the fine tuning of LSPR of Au–Ag nanocages through the selective etching by oxygen and water-soluble thiol (Cho et al., 2009), and also employed this strategy to measure the concentration of H_2_O_2_ (Zhang et al., 2010). Lin and Wang et al. prepared a new type of Au–Ag nanorods with an Au seed at one tip of the rod, and monitored the selective etching of Ag from another tip for hypochlorite detection (Li et al., 2019a). The strategy of etching surface Ag of Au–Ag nanoparticles with different shapes (e.g., spheres, rods, and bipyramids) were also applied for sensing of a variety of different analytes such as cyanide (Zeng et al., 2014; Li Y. et al., 2016), iodide (Qi et al., 2017), Hg^2+^/Pb^2+^ (Yang et al., 2015; Xing et al., 2018), permanganate (Ye et al., 2018), benzoyl peroxide (Lin et al., 2018), MicroRNA (Gu et al., 2017) and proteins (Liu H. et al., 2015; Liu W. et al., 2015).

## Conclusion and Perspectives

Great progress in synthesis and characterization of bimetallic nanocrystals in recent years gives better insight into the relationship between their composition/morphology and LSPR properties. Understanding of the “rules” involving in their formation is of great significance to design desired structures and LSPR for sensing applications. To this end, improved theoretical models in combination with systematic experimental studies are needed to achieve the “design” instead of “explore” the well-defined bimetallic nanostructures with good plasmonic performances. Moreover, advanced *in-situ* monitoring technologies such as liquid transmission electron microscopy and dark-field scattering microscopy/spectroscopy will play important roles in real-time observing the formation or evolution of the bimetallic nanostructures (Shan et al., 2018; Ye et al., 2018; Zhou et al., 2018). For sensing purpose, bimetallic sensors take advantage of their component and spatial distribution of different metals. Thus, manipulating the bimetallic nanoprobes and precise tuning their LSPR signal can provide improved sensitivity and reproducibility of the sensor. Although a variety of plasmonic sensors or analytical methodologies have been established based on the LSPR peak shift of bimetallic nanostructures, further investigations on the detection mechanism involved and their practical applications are still challenging. Specificity may be another question that needs to be resolved before the bimetallic plasmonic sensors walking from laboratories to application. Surface modification or capping of the bimetallic nanoprobes with any ligands may achieve the specific recognitions between ligands and target analytes (e.g., antigen-antibody, aptamer-target molecule, and host-guest molecule). In addition, exploring the LSPR tunability of Cu (Chen et al., 2019) and other low-cost metals as well as improving their chemical stability will be of great benefit to reduce the cost of bimetallic plasmonic sensors in the future.

## Author Contributions

YM collected all the literatures, organized the figures and wrote part of the synthetic approaches to bimetallic nanostructures. YW wrote other sections and oversaw the project.

## Conflict of Interest

The authors declare that the research was conducted in the absence of any commercial or financial relationships that could be construed as a potential conflict of interest.

## References

[B1] Anker J. N. Hall W. P. Lyandres O. Shah N. C. Zhao J. Van duyne R. P. (2008). Biosensing with plasmonic nanosensors. Nat. Mater. 7, 442–453. 10.1038/nmat2162 18497851

[B2] Bai Y. Gao C. Yin Y. (2017). Fully alloyed Ag/Au nanorods with tunable surface plasmon resonance and high chemical stability. Nanoscale 9, 14875–14880. 10.1039/c7nr06002e 28975172

[B3] Baldi A. Narayan T. C. Koh A. L. Dionne J. A. (2014). *In situ* detection of hydrogen-induced phase transitions in individual palladium nanocrystals. Nat. Mater. 13, 1143–1148. 10.1038/nmat4086 25194700

[B4] Cargnello M. Agarwal R. Klein D. R. Diroll B. T. Agarwal R. Murray C. B. (2015). Uniform bimetallic nanocrystals by high temperature seed mediated colloidal synthesis and their catalytic properties for semiconducting nanowire growth. Chem. Mater. 27, 5833–5838. 10.1021/acs.chemmater.5b02900

[B5] Cattaruzza E. Battaglin G. Gonella F. Polloni R. Scremin B. F. Mattei G. (2007). Au-Cu nanoparticles in silica glass as composite material for photonic applications. Appl. Surf. Sci. 254, 1017–1021. 10.1016/j.apsusc.2007.07.158

[B6] Chen H. Shao L. Li Q. Wang J. (2013). Gold nanorods and their plasmonic properties. Chem. Soc. Rev. 42, 2679–2724. 10.1039/c2cs35367a 23128995

[B7] Chen H. Wang F. Li K. Woo K. C. Wang J. Li Q.. (2012). Plasmonic percolation: plasmon manifested dielectric-to-metal transition. ACS Nano 6, 7162–7171. 10.1021/nn302220y 22757659

[B8] Chen J. Feng J. Yang F. Aleisa R. Zhang Q. Yin Y. (2019). Space-confined seeded growth of Cu nanorods with strong surface plasmon resonance for photothermal actuation. Angew. Chem. Int. Ed. 131, 9376–9382. 10.1002/ange.201904828 31062923

[B9] Chen J. McLellan J. M. Siekkinen A. Xiong Y. Li Z. Xia Y. (2006). Facile synthesis of gold-silver nanocages with controllable pores on the surface. J. Am. Chem. Soc. 128, 14776–14777. 10.1021/ja066023g 17105266 PMC2532083

[B10] Chen J. Wiley B. McLellan J. Xiong Y. Li Z. Xia Y. (2005). Optical properties of Pd–Ag and Pt–Ag nanoboxes synthesized via galvanic replacement reactions. Nano Lett. 5, 2058–2062. 10.1021/nl051652u 16218737

[B11] Chen L. Li H. He H. Wu H. Jin Y. (2015). Smart plasmonic glucose nanosensors as generic theranostic agents for rapid, targeting-free cancer-cell screening and killing. Anal. Chem. 87, 6868–6874. 10.1021/acs.analchem.5b01260 26027697

[B12] Chiu C.-Y. Yang M.-Y. Lin F.-C. Huang J.-S. Huang M. H. (2014). Facile synthesis of Au–Pd core–shell nanocrystals with systematic shape evolution and tunable size for plasmonic property examination. Nanoscale 6, 7656–7665. 10.1039/c4nr01765j 24898776

[B13] Cho E. C. Cobley C. M. Rycenga M. Xia Y. (2009). Fine tuning the optical properties of Au–Ag nanocages by selectively etching Ag with oxygen and a water-soluble thiol. J. Mater. Chem. 19, 6317–6320. 10.1039/b901817d 19829745 PMC2760974

[B14] Cortie M. B. McDonagh A. M. (2011). Synthesis and optical properties of hybrid and alloy plasmonic nanoparticles. Chem. Rev. 111, 3713–3735. 10.1021/cr1002529 21235212

[B15] DeSantis C. J. Skrabalak S. (2012). Size-controlled synthesis of Au/Pd octopods with high refractive index sensitivity. Langmuir 28, 9055–9062. 10.1021/la3002509 22428850

[B16] DeSantis C. J. Skrabalak S. (2013). Core values: elucidating the role of seed structure in the synthesis of symmetrically branched nanocrystals*. J. Am. Chem. Soc* 135, 10–13. 10.1021/ja308456w 23270415

[B17] DeSantis C. J. Weiner R. G. Randmilovic A. Bower M. M. Skrabalak S. E. (2013). Seeding bimetallic nanostructures as a new class of plasmonic colloids. J. Phys. Chem. Lett. 4, 3072–3082. 10.1021/jz4011866

[B18] Gao C. Hu Y. Wang M. Chi M. Yin Y. (2014). Fully alloyed Ag/Au nanospheres: combining the plasmonic property of Ag with the stability of Au. J. Am. Chem. Soc. 136, 7474–7479. 10.1021/ja502890c 24821567

[B19] Gao Z. Deng K. Wang X. Miró M. Tang D. (2014). A high-resolution colorimetric assay for rapid visual readout of phosphatase activity based on gold/silver core/shell nanorod. ACS Appl. Mater. Interfaces 6, 18243–18250. 10.1021/am505342r 25244147

[B20] Gilroy K. D. Rudistskiy A. Peng H.-C. Qin D. Xia Y. (2016). Bimetallic nanocrystals: syntheses, properties, and applications. Chem. Rev. 116, 10414–10472. 10.1021/acs.chemrev.6b00211 27367000

[B21] González E. Arbiol J. Puntes V. F. (2011). Carving at the nanoscale: sequential galvanic exchange and kirkendall growth at room temperature. Science 334, 1377–1380. 10.1126/science.1212822 22158813

[B22] Gu Y. Song J. Li M.-X. Zhang T. Zhao W. Xu J.-J. (2017). Ultra-sensitive microRNA sssay via surface plasmon resonance responses of Au@Ag nanorods etching. Anal. Chem. 89, 10585–10591. 10.1021/acs.analchem.7b02920 28872300

[B23] Habas S. E. Lee H. Rodmilovic V. Somorjai G. A. Yang P. (2007). Shaping binary metal nanocrystals through epitaxial seeded growth. Nat. Mater. 6, 692–697. 10.1038/nmat1957 17618289

[B24] Haes A. J. Van Duyne R. P. (2004). A unified view of propagating and localized surface plasmon resonance biosensors. Anal. Bioanal. Chem. 379, 920–930. 10.1007/s00216-004-2708-9 15338088

[B25] Hajfathalian M. Gilroy K. D. Yaghoubzade A. Sundar A. Tan T. Hughes R. A. (2015). Photocatalytic enhancements to the reduction of 4-nitrophenol by resonantly excited triangular gold-copper nanostructures. J. Phys. Chem. C 119, 17308–17315. 10.1021/acs.jpcc.5b04618

[B26] Hao J. Xiong B. Cheng X. He Y. Yeung E. S. (2014). High-throughput sulfide sensing with colorimetric analysis of single Au–Ag core–shell nanoparticles. Anal. Chem. 86, 4663–4667. 10.1021/ac500376e 24809220

[B27] He H. Xu X. Wu H. Jin Y. (2012). Enzymatic plasmonic engineering of Ag/Au bimetallic nanoshells and their use for sensitive optical glucose sensing. Adv. Mater. 24, 1736–1740. 10.1002/adma.201104678 22388952

[B28] He H. Xu X. Wu H. Zhai Y. Jin Y. (2013). In situ nanoplasmonic probing of enzymatic activity of monolayer confined glucose oxidase on colloidal nanoparticles. Anal. Chem. 85, 4546–4553. 10.1021/ac4001805 23531235

[B29] Henglein A. (2000). Preparation and optical absorption spectra of Au_core_Pt_shel_l and Pt_core_Au_shell_ colloidal nanoparticles in aqueous solution. J. Phys. Chem. B 104, 2201–2203. 10.1021/jp994300i

[B30] Hong X. Wang D. Cai S. Rong H. Li Y. (2012). Single-crystalline octahedral Au–Ag nanoframes. J. Am. Chem. Soc. 134, 18165–18168. 10.1021/ja3076132 23088493

[B31] Hou S. Hu X. Wen T. Liu W. Wu X. (2013). Core–shell noble metal nanostructures templated by gold nanorods. Adv. Mater. 25, 3857–3862. 10.1002/adma.201301169 24048971

[B32] Huang X. Tang S. Mu X. Dai Y. Chen G. Zhou Z. (2011). Freestanding palladium nanosheets with plasmonic and catalytic properties. Nat. Nanotech. 6, 28–32. 10.1038/nnano.2010.235 21131956

[B33] Jain P. K. Huang X. Sayed E. I. Sayed M. A. (2007). Review of some interesting surface plasmon resonance-enhanced properties of noble metal nanoparticles and their applications to biosystems. Plasmonics 2, 107–118. 10.1007/s11468-007-9031-1

[B34] Jain P. K. Huang X. Sayed E. I. Sayed M. A. (2008). Noble metals on the nanoscale: optical and photothermal properties and some applications in imaging, sensing, biology, and medicine. Acc. Chem. Res. 4, 21578–21586. 10.1021/ar7002804 18447366

[B35] Jiang R. Chen H. Shao L. Li Q. Wang J. (2012). Unraveling the evolution and nature of the plasmons in (Au core)–(Ag shell) nanorods. Adv. Mater. 24, 200–207. 10.1002/adma.201201896 22714684

[B36] Jin M. Zhang H. Wang J. Zhong X. Lu N. Li Z.. (2012). Copper can still be epitaxially deposited on palladium nanocrystals to generate core-shell nanocubes despite their large lattice mismatch. ACS Nano 6, 2566–2573. 10.1021/nn2050278 22303890

[B37] Jin Y. (2014). Multifunctional compact hybrid Au nanoshells: a new generation of nanoplasmonic probes for biosensing, imaging, and controlled release. Acc. Chem. Res. 47, 138–148. 10.1021/ar400086e 23992824

[B38] Kahraman M. Aydin Ö. Çulha M. (2009). Oligonucleotide-mediated Au–Ag core–shell nanoparticles. Plasmonics 4, 293–301. 10.1007/s11468-009-9105-3

[B39] Khanal B. P. Zubarev E. R. (2009). Polymer-functionalized platinum-On-gold bimetallic nanorods. Angew. Chem. Int. Ed. 121, 7020–7023. 10.1002/anie.200903524 19681086

[B40] Kim D. Y. Resasco J. Yu Y. Asiri A. M. Yang P. (2014). Synergistic geometric and electronic effects for electrochemical reduction of carbon dioxide using gold–copper bimetallic nanoparticles. Nat. Commun. 5:4948. 10.1038/ncomms5948 25208828

[B41] Kim M. J. Na H.-J. Lee K. C. Yoob E. A. Lee M. (2003). Preparation and characterization of Au–Ag and Au–Cu alloy nanoparticles in chloroform. J. Mater. Chem. 13, 1789–1792. 10.1039/b304006m

[B42] Lahiri D. Bunker B. Mishra B. Zhang Z. Meisel D. Doudna C. M. (2005). Bimetallic Pt–Ag and Pd–Ag nanoparticles. J. Appl. Phys. 97:094304 10.1063/1.1888043

[B43] Lei G. Gao P. Liu H. Huang C. (2015). Real-time scattered light dark-field microscopic imaging of the dynamic degradation process of sodium dimethyldithiocarbamate. Nanoscale 7, 20709–20716. 10.1039/c5nr05838d 26601853

[B44] Li J. Zheng Y. Zeng J. Xia Y. (2012). Controlling the size and morphology of Au@Pd core–shell nanocrystals by manipulating the kinetics of seeded growth. Chem. Eur. J. 18, 8150–8156. 10.1002/chem.201200823 22615213

[B45] Li L. Zhang P. Fu W. Yang M. Wang Y. (2018). Use of seed-mediated growth of bimetallic nanorods as a knob for antioxidant assay. Sens. Actuat. B Chem. 276, 158–165. 10.1016/j.snb.2018.08.104

[B46] Li X. Lin X. Lin S. Sun X. Gao D. Liu B. (2019a). Au nanospheres@Ag nanorods for wide linear range colorimetric determination of hypochlorite. ACS Appl. Nano Mater. 2, 3161–3168. 10.1021/acsanm.9b00475

[B47] Li X. Zhang W. Cui W. Li J. Sun Y. Jiang G. (2019b). Reactant activation and photocatalysis mechanisms on Bi-metal@Bi_2_GeO_5_ with oxygen vacancies: a combined experimental and theoretical investigation. Chem. Eng. J. 370, 1366–1375. 10.1016/j.cej.2019.04.003

[B48] Li X. Zhang W. Cui W. Sun Y. Jiang G. Zhang Y. (2018). Bismuth spheres assembled on graphene oxide: directional charge transfer enhances plasmonic photocatalysis and *in situ* DRIFTS studies. Appl. Catal. B Environ. 221, 482–489. 10.1016/j.apcatb.2017.09.046

[B49] Li X. Zhang W. Li J. Jiang G. Zhou Y. Lee S. C. (2019c). Transformation pathway and toxic intermediates inhibition of photocatalytic NO removal on designed Bi metal@defective Bi_2_O_2_SiO_3_. Appl. Catal. B Environ. 241, 187–195. 10.1016/j.apcatb.2018.09.032

[B50] Li X. Zhu J. Wei B. (2016). Hybrid nanostructures of metal/two-dimensional nanomaterials for plasmon-enhanced applications. Chem. Soc. Rev. 45, 3145–3187. 10.1039/c6cs00195e 27048993

[B51] Li Y. Wang Q. Zhou X. Wen C. Yu J. Han X. (2016). A convenient colorimetric method for sensitive and specific detection of cyanide using Ag@Au core-shell nanoparticles. Sens. Actuat. B Chem. 228, 366–372. 10.1016/j.snb.2016.01.022

[B52] Lin T. Zhang M. Xu F. Wang X. Xu Z. Guo L. (2018). Colorimetric detection of benzoyl peroxide based on the etching of silver nanoshells of Au@Ag nanorods. Sens. Actuat. B Chem. 261, 379–384. 10.1016/j.snb.2018.01.172

[B53] Link S. Wang Z. Sayed M. A. (1999). Alloy formation of gold-silver nanoparticles and the dependence of the plasmon absorption on their composition. J. Phys. Chem. B 103, 3529–3533. 10.1021/jp990387w

[B54] Liu H. Liu T. Zhang L. Han L. Gao C. Yin Y. (2015). Etching-free epitaxial growth of gold on silver nanostructures for high chemical stability and plasmonic activity. Adv. Funct. Mater. 25, 5435–5443. 10.1002/adfm.201502366

[B55] Liu N. Tang M. Hentschel M. Giessen H. Alivisatos A. P. (2011c). Nanoantenna-enhanced gas sensing in a single tailored nanofocus. Nat. Mater. 10, 631–636. 10.1038/nmat3029 21572410

[B56] Liu W. Hou S. Yan J. Zhang H. Ji Y. Wu X. (2015). Quantification of proteins using enhanced etching of Ag coated Au nanorods by the Cu^2+^/ bicinchoninic acid pair with improved sensitivity. Nanoscale 8, 780–784. 10.1039/c5nr07924a 26669539

[B57] Liu Y. Chi M. Mazumder V. More K. Soled S. Henao J. (2011a). Composition-controlled synthesis of bimetallic PdPt nanoparticles and their electro-oxidation of methanol. Chem. Mater. 23, 4199–4203. 10.1021/cm2014785

[B58] Liu Y. Huang C. Z. (2013a). Digitized single scattering nanoparticles for probing molecular binding. Chem. Commun. 49, 8262–8264. 10.1039/c3cc43605e 23925066

[B59] Liu Y. Huang C. Z. (2013b). Real-time dark-field scattering microscopic monitoring of the *in situ* growth of single Ag@Hg nanoalloys. ACS Nano 7, 11026–11034. 10.1021/nn404694e 24279755

[B60] Liu Y. Ling J. Huang C. Z. (2011b). Individually color-coded plasmonic nanoparticles for RGB analysis. Chem. Commun. 47, 8121–8123. 10.1039/c1cc11503k 21687902

[B61] Lohse S. E. Burrows N. D. Scarabelli L. Liz-Marzan L. M. Murphy C. J. (2014). Anisotropic noble metal nanocrystal growth: the role of halides. Chem. Mater. 26, 34–43. 10.1021/cm402384j

[B62] Ma Y. Li W. Cho E. C. Li Z. Yu T. Zeng J.. (2010). Au@Ag core-shell nanocubes with finely tuned and well-controlled sizes, shell thicknesses, and optical properties. ACS Nano 4, 6725–6734. 10.1021/nn102237c 20964400 PMC2997519

[B63] Mallin M. P. Murphy C. J. (2002). Solution-phase synthesis of sub-10 nm Au–Ag alloy nanoparticles. ACS Nano 2, 1235–1237. 10.1021/nl025774n

[B64] Mandal S. Mandale A. B. Sastry M. (2004). Keggin ion-mediated synthesis of aqueous phase-pure Au@Pd and Au@Pt core–shell nanoparticles. J. Mater. Chem. 14, 2868–2871. 10.1039/b409033k

[B65] Mashtalir O. Naguib M. Mochalin V. N. Agnese Y. D. Heon M. Barsoum M. W. (2013). Intercalation and delamination of layered carbides and carbonitrides. Nat. Commun. 4:1716. 10.1038/ncomms2664 23591883

[B66] Mayer K. M. Hafner J. H. (2011). Localized surface plasmon resonance sensors. Chem. Rev. 111, 3828–3857. 10.1021/cr100313v 21648956

[B67] Mayer M. Scarabelli L. March K. Altantzis T. Tebbe M. Kociak M.. (2015). Controlled living nanowire growth: precise control over the morphology and optical properties of AgAuAg bimetallic nanowires. Nano Lett. 15, 5427–5437. 10.1021/acs.nanolett.5b01833 26134470 PMC4538453

[B68] McFarland A. D. Van Duyne R. P. (2003). Single silver nanoparticles as real-time optical sensors with zeptomole sensitivity. Nano Lett. 3, 1057–1062. 10.1021/nl034372s

[B69] Métraux G. S. Cao Y. C. Jin R. C. Mirkin C. A. (2003). Triangular nanoframes made of gold and silver. Nano Lett. 3, 519–522. 10.1021/nl034097+

[B70] Moskovits M. Srnová-Šloufová I. Vlčková B. (2002). Bimetallic Ag–Au nanoparticles: extracting meaningful optical constants from the surface-plasmon extinction spectrum. J. Chem. Phys. 116, 10435–10446. 10.1063/1.1449943

[B71] Motl N. E. Ewusi-Annan E. Sines I. T. Jensen L. Schaak R. E. (2010). Au-Cu alloy nanoparticles with tunable compositions and plasmonic properties: experimental determination of composition and correlation with theory. J. Phys. Chem. C 114, 19263–19269. 10.1021/jp107637j

[B72] Murphy C. J. Sau T. K. Gole A. M. Orendorff C. J. Gao J. X. Gou L. F.. (2005). Anisotropic metal nanoparticles: synthesis, assembly, and optical applications. J. Phys. Chem. B 109, 13857–13870. 10.1021/jp0516846 16852739

[B73] Qi Y. Zhu J. Li J.-J. Zhao J.-W. (2017). Multi-mode optical detection of iodide based on the etching of silver-coated gold nanobipyramids. Sens. Actuat. B Chem. 253, 612–620. 10.1016/j.snb.2017.06.180

[B74] Rodríguez-Lorenzo L. Rica R. D. L. Álvarez-Puebla R. A. Liz-Marzán L. M. Steven M. M. (2012). Plasmonic nanosensors with inverse sensitivity by means of enzyme-guided crystal growth. Nat. Mater. 11, 604–607. 10.1038/nmat3337 22635043

[B75] Rycenga M. Cobley C. M. Zeng J. Li W. Moran C. H. Zhang Q.. (2011). Controlling the synthesis and assembly of silver nanostructures for plasmonic applications. Chem. Rev. 111, 3669–3712. 10.1021/cr100275d 21395318 PMC3110991

[B76] Shan H. Gao W. Xiong Y. Shi F. Yan Y. Ma Y.. (2018). Nanoscale kinetics of asymmetrical corrosion in core-shell nanoparticles. Nat. Commun. 9:1011. 10.1038/s41467-018-03372-z 29520056 PMC5843659

[B77] Shore M. S. Wang J. Johnston-Peck A. C. Oldenburg A. L. Tracy J. B. (2010). Synthesis of Au(core)/Ag(shell) nanoparticles and their conversion to AuAg alloy nanoparticles. Small 7, 230–234. 10.1002/smll.201001138 21213387

[B78] Skrabalak S. E. Au L. Li X. Xia Y. (2007a). Facile synthesis of Ag nanocubes and Au nanocages. Nat. Protoc 2, 2182–2190. 10.1038/nprot.2007.326 17853874

[B79] Skrabalak S. E. Chen J. Au L. Lu X. Li X. Xia Y. (2007b). Gold nanocages for biomedical applications. Adv. Mater. 19, 3177–3184. 10.1002/adma.200701972 18648528 PMC2480527

[B80] Skrabalak S. E. Chen J. Sun Y. Lu X. Au L. Cobley C. M.. (2008). Gold nanocages: synthesis, properties, and applications. Acc. Chem. Res. 41, 1587–1595. 10.1021/ar800018v 18570442 PMC2645935

[B81] Smetana A. B. Klabunde K. J. Sorensen C. M. Ponce A. A. Mwale B. (2006). Low-temperature metallic alloying of copper and silver nanoparticles with gold nanoparticles through digestive ripening. J. Phys. Chem. B 110, 2155–2158. 10.1021/jp0539932 16471798

[B82] Sun Y. Mayers B. T. Xia Y. (2002a). Template-engaged replacement reaction: a one-step approach to the large-scale synthesis of metal nanostructures with hollow interiors. Nano Lett. 2, 481–485. 10.1021/nl025531v

[B83] Sun Y. Wiley B. Li Z.-Y. Xia Y. (2004). Synthesis and optical properties of nanorattles and multiple-walled nanoshells/nanotubes made of metal alloys. J. Am. Chem. Soc. 126, 9399–9406. 10.1021/ja048789r 15281832

[B84] Sun Y. Xia Y. (2002). Shape-controlled synthesis of gold and silver nanoparticles. Science 298, 2176–2179. 10.1126/science.1077229 12481134

[B85] Sun Y. Xia Y. (2004a). Multiple-walled nanotubes made of metals. Adv. Mater. 16, 264–268. 10.1002/adma.200305780

[B86] Sun Y. Xia Y. (2004b). Mechanistic study on the replacement reaction between silver nanostructures and chloroauric acid in aqueous. J. Am. Chem. Soc. 126, 3892–3901. 10.1021/ja039734c 15038743

[B87] Sun Y. Yin Y. Mayers B. T. Herricks T. Xia Y. (2002b). Uniform silver nanowires synthesis by reducing AgNO_3_ with ethylene glycol in the presence of seeds and poly(vinyl pyrrolidone). Chem. Mater. 14, 4736–4745. 10.1021/cm020587b

[B88] Tang M. L. Liu N. Dionne J. A. Alivisatos A. P. (2011). Observations of shape-dependent hydrogen uptake trajectories from single nanocrystals. J. Am. Chem. Soc. 133, 13220–13223. 10.1021/ja203215b 21793566

[B89] Tao A. R. Habas S. Yang P. (2008). Shape control of colloidal metal nanocrystals. Small 4, 310–325. 10.1002/smll.200701295

[B90] Tsao Y.-C. Rej S. Chiu C.-Y. Huang M. H. (2014). Aqueous phase synthesis of Au-Ag core-shell nanocrystals with tunable shapes and their optical and catalytic properties. J. Am. Chem. Soc. 136, 396–404. 10.1021/ja410663g 24341355

[B91] Wang C. Asrtuc D. (2014). Nanogold plasmonic photocatalysis for organic synthesis and clean energy conversion. Chem. Soc. Rev. 43, 7188–7216. 10.1039/c4cs00145a 25017125

[B92] Wang C. Yin H. Chan R. Peng S. Dai S. Sun S. (2009). One-pot synthesis of oleylamine coated AuAg alloy NPs and their catalysis for CO oxidation. Chem. Mater. 21, 433–435. 10.1021/cm802753j

[B93] Wang J. Fossey J. S. Li M. Xie T. Long Y.-T. (2016). Real-time plasmonic monitoring of single gold amalgam nanoalloy electrochemical formation and stripping. ACS Appl. Mater. Interfaces 8, 8305–8314. 10.1021/acsami.6b01029 26942394

[B94] Wang Y. Fu W. Hu X. (2015a). Facile synthesis of high-purity single-twinned Au nanocrystals through manipulating reaction kinetics. CrystEngComm. 17, 6636–6640. 10.1039/c5ce01000d

[B95] Wang Y. Wan D. Xie S. Xia X. Huang C. Z. Xia Y. (2013a). Synthesis of silver octahedra with controlled sizes and optical properties via seed-mediated growth. ACS Nano 7, 4586–4594. 10.1021/nn401363e 23631674

[B96] Wang Y. Zeng Y. Fu W. Zhang P. Li L. Ye C.. (2018b). Seed-mediated growth of Au@Ag core-shell nanorods for the detection of ellagic acid in whitening cosmetics. Anal. Chim. Acta 1002, 97–104. 10.1016/j.aca.2017.11.067 29306418

[B97] Wang Y. Zhang P. Fu W. Zhao Y. (2018a). Morphological control of nanoprobe for colorimetric antioxidant detection. Biosens. Bioelectron. 122, 183–188. 10.1016/j.bios.2018.09.058 30265968

[B98] Wang Y. Zhang P. Mao X. Fu W. Liu C. (2016). Seed-mediated growth of bimetallic nanoparticles as an effective strategy for sensitive detection of vitamin C. Sens. Actuat. B Chem. 231, 95–101. 10.1016/j.snb.2016.03.010

[B99] Wang Y. Zheng Y. Huang C. Z. Xia Y. (2013b). Synthesis of Ag nanocubes 18–32 nm in edge length: the effects of polyol on reduction kinetics, size control, and reproducibility. J. Am. Chem. Soc. 135, 1941–1951. 10.1021/ja311503q 23317148 PMC3576731

[B100] Wang Y. Zou H. Y. Huang C. Z. (2015b). Real-time monitoring of oxidative etching on single Ag nanocube via light-scattering dark-field microscopic imaging. Nanoscale 7, 15209–15213. 10.1039/c5nr04234h 26316076

[B101] Willets K. A. Van Duyne R. P. (2007). Localized surface plasmon resonance spectroscopy and sensing. Annu. Rev. Phys. Chem. 58, 267–297. 10.1146/annurev.physchem.58.032806.104607 17067281

[B102] Wy Y. Lee S. Wi D. H. Han S. W. (2018). Colloidal clusters of bimetallic core–shell nanoparticles for enhanced sensing of hydrogen in aqueous solution. Part. Part. Syst. Charact. 35:1700380 10.1002/ppsc.201700380

[B103] Xia C. He W. Gao P. F. Wang J. R. Cao Z. M. Li Y. F.. (2020). Nanofabrication of hollowed-out Au@AgPt core-frames via selective carving of silver and deposition of platinum. Chem. Commun. 56, 2945–2948. 10.1039/c9cc09573j 32040110

[B104] Xia X. Figueroa-Cosme L. Tao J. Peng H.-C. Niu G. Zhu Y.. (2014). Facile synthesis of iridium nanocrystals with well-controlled facets using seed-mediated growth. J. Am. Chem. Soc. 136, 10878–10881. 10.1021/ja505716v 25058427

[B105] Xia X. Wang Y. Ruditskiy A. Xia Y. (2013). 25th anniversary article: galvanic replacement: a simple and versatile route to hollow nanostructures with tunable and well-controlled properties. Adv. Mater. 25, 6313–6333. 10.1002/adma.201302820 24027074

[B106] Xia Y. Gilroy K. D. Peng H.-C. Xia X. (2016). Seed-mediated growth of colloidal metal nanocrystals. Angew. Chem. Int. Ed. 56, 60–95. 10.1002/anie.201604731 27966807

[B107] Xia Y. Xiong Y. Lim B. Skrabalak S. E. (2009). Shape-controlled synthesis of metal nanocrystals: simple chemistry meets complex physics? Angew. Chem. Int. Ed. 48, 60–103. 10.1002/anie.200802248 19053095 PMC2791829

[B108] Xie S. Jin M. Tao J. Wang Y. Xie Z. Zhu Y.. (2012a). Synthesis and characterization of Pd@M_x_Cu_1−x_ (M=Au, Pd, and Pt) nanocages with porous walls and a yolk–shell structure through galvanic replacement reactions. Chem. Eur. J. 18, 14974–14980. 10.1002/chem.201202477 23108763

[B109] Xie S. Lu N. Xie Z. Wang J. Kim M. J. Xia Y. (2012b). Synthesis of Pd-Rh core–frame concave nanocubes and their conversion to Rh cubic nanoframes by selective etching of the Pd cores. Angew. Chem. Int. Ed. 51, 10266–10270. 10.1002/anie.201206044 22968993

[B110] Xie S. Peng H.-C. Lu N. Wang J. Kim M. J. Xie Z.. (2013). Confining the nucleation and overgrowth of Rh to the {111} facets of Pd nanocrystal seeds: the roles of capping agent and surface diffusion. J. Am. Chem. Soc. 135, 16658–16667. 10.1021/ja408768e 24116876

[B111] Xing T.-Y. Zhao J. Weng G.-J. Li J.-J. Zhu J. Zhao J.-W. (2018). Synthesis of dual-functional Ag/Au nanoparticles based on decreased cavitating rate under alkaline condition and the colorimetric detection of mercury(II) and lead(II). J. Mater. Chem. C. 6, 7557–7567. 10.1039/C8TC01867G

[B112] Xiong Y. Xia Y. (2007). Shape-controlled synthesis of metal nanostructures: the case of palladium. Adv. Mater. 19, 3385–3391. 10.1002/adma.200701301

[B113] Xu S. Ouyang W. Xie P. Lin Y. Qiu B. Lin Z.. (2017). Highly uniform gold nanobipyramids for ultrasensitive colorimetric detection of influenza virus. Anal. Chem. 89, 1617–1623. 10.1021/acs.analchem.6b03711 28208287

[B114] Yang R. Song D. Wang C. Zhu A. Xiao R. Liu J. (2015). Etching of unmodified Au@Ag nanorods: a tunable colorimetric visualization for rapid and high selective detection of Hg^2+^. RSC Adv. 5, 102542–102549. 10.1039/c5ra19627b

[B115] Ye Z. Weng R. Ma Y. Wang F. Liu H. Wei L.. (2018). Label-free single-particle colorimetric detection of permanganate by GNPs@Ag core-shell nanoparticle with dark-field optical microscopy. Anal. Chem. 90, 13044–13050. 10.1021/acs.analchem.8b04024 30289245

[B116] Zeng J. Gao Y. Chen J. Wang X. Yu J. Yu B.. (2014). Au@Ag core/shell nanoparticles as colorimetric probes for cyanide sensing. Nanoscale 6, 9939–9943. 10.1039/c4nr02560a 25054637

[B117] Zeng J. Zhu C. Tao J. Jin M. Zhang H. Li Z.-Y.. (2012). Controlling the nucleation and growth of silver on palladium nanocubes by manipulating the reaction kinetics. Angew. Chem. Int. Ed. 51, 2354–2358. 10.1002/anie.201107061 22105984

[B118] Zhang C. Yin A.-X. Jiang R. Rong J. Dong L. Zhao T.. (2013). Time-temperature indicator for perishable products based on kinetically programmable Ag overgrowth on Au nanorods. ACS Nano 7, 4561–4568. 10.1021/nn401266u 23627773

[B119] Zhang H. Jin M. Wang J. Li W. Camargo P. H. C. Kim M. J.. (2011). Synthesis of Pd-Pt bimetallic nanocrystals with a concave structure through a bromide-induced galvanic replacement reaction. J. Am. Chem. Soc. 133, 6078–6089. 10.1021/ja201156s 21438596

[B120] Zhang P. Wang L. Zeng J. Tan J. Long Y. Wang Y. (2020). Colorimetric captopril assay based on oxidative etching-directed morphology control of silver nanoprisms. Microchim. Acta 187:107. 10.1007/s00604-019-4071-8 31915936

[B121] Zhang Q. Cobley C. M. Zeng J. Wen L.-P. Chen J. Xia Y. (2010). Dissolving Ag from Au-Ag Alloy Nanoboxes with H_2_O_2_: a method for both tailoring the optical properties and measuring the H_2_O_2_ concentration. J. Phys. Chem. C 114, 6396–6400. 10.1021/jp100354z PMC2873216 20495675

[B122] Zhang Q. Xie J. Lee J. Y. Zhang J. Boothroyd C. (2008). Synthesis of Ag@AgAu metal core/alloy shell bimetallic nanoparticles with tunable shell compositions by a galvanic replacement reaction. Small 4, 1067–1071. 10.1002/smll.200701196 18651712

[B123] Zhang S. Metin Ö. Su D. Sun S. (2013). Monodisperse AgPd alloy nanoparticles and their superior catalysis for the dehydrogenation of formic acid. Angew. Chem. Int. Ed. 52, 3681–3684. 10.1002/anie.201300276 23426846

[B124] Zhang W. Goh H. Y. J. Firdoz S. Lu X. (2013). Growth of Au@Ag core–shell pentatwinned nanorods: tuning the end facets. Chem. Eur. J 19, 12732–12738. 10.1002/chem.201301753 23934938

[B125] Zhao Y. Gao X.-Y. Wang H. Wang J. Zhou J. Zhao W. (2019). Ultra-sensitive detection of microRNA via Au@Ag “nanosnowman”. Anal. Chem. 91, 15988–15992. 10.1021/acs.analchem.9b04715 31718153

[B126] Zhou J. Yang T. He W. Pan Z. Y. Huang C. Z. (2018). A galvanic exchange process visualized on single silver nanoparticles via dark-field microscopic imaging. Nanoscale 10, 12805–12812. 10.1039/c8nr01879k 29947404

